# Supplementary material for: Low Neutrophil-to-Lymphocyte Ratio Combined with High Intraepithelial CD8+ Tumour-Infiltrating Lymphocytes Within the Tumour Microenvironment Is a Prominent Prognostic Factor in Advanced Epithelial Ovarian Cancer
Source: Cancers (Basel). 2025 Dec 6;17(24):3904. doi: 10.3390/cancers17243904 (PMC12730443; doi:10.3390/cancers17243904)
Supplement: Supplementary file 1 [file cancers-17-03904-s001.zip › cancers-3987410-supplementary.pdf]

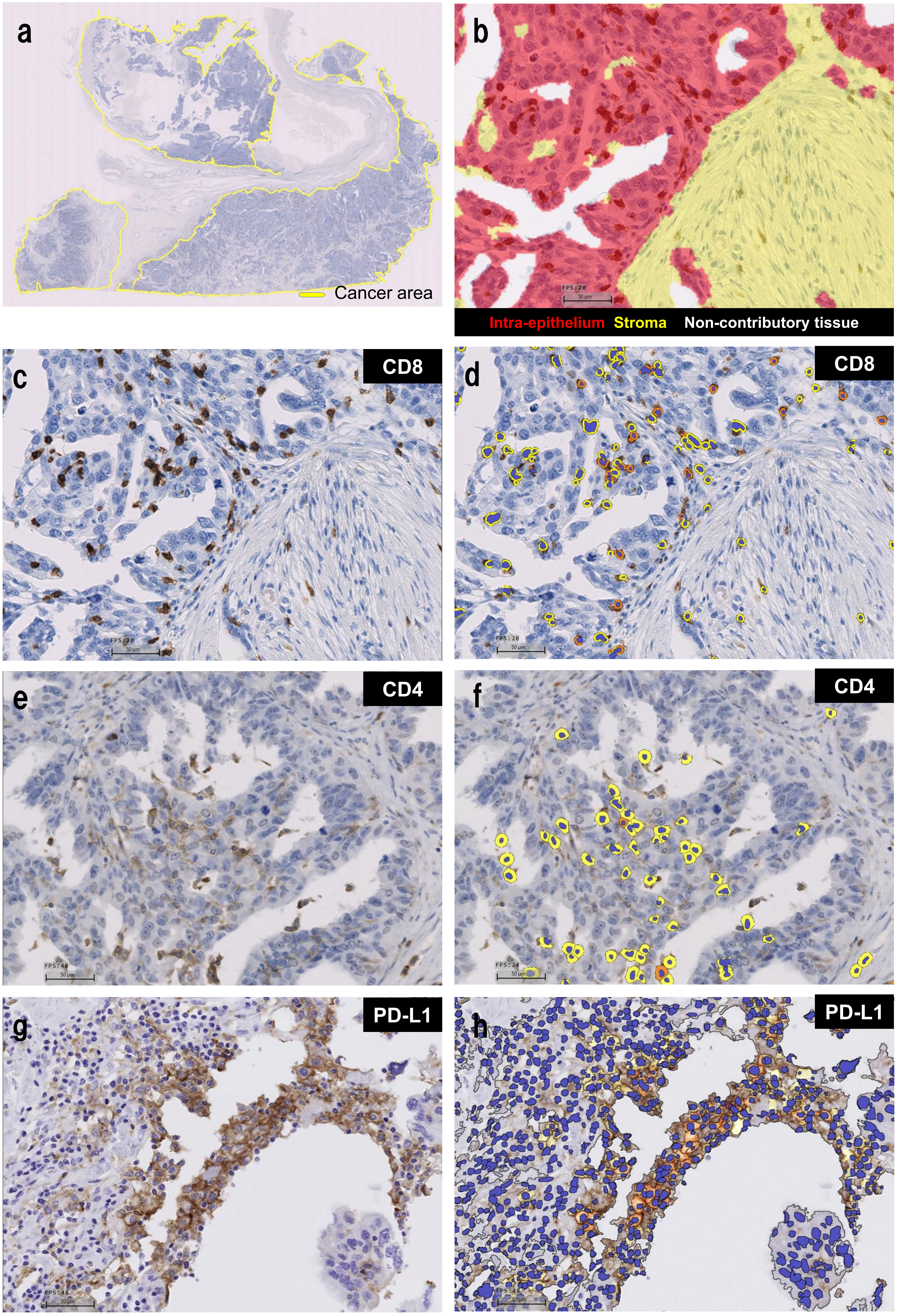

Figure S1:
Example of the digital TILs quantification with the HALO® image analysis platform. **a.** The annotated cancer area. **b.** At greater magnification the software was trained for tissue classification. Tree classes where defined: intra-epithelium, stroma, and non-contributory tissue. **c, e, g.** High-grade serous carcinoma specimen stained with CD8, CD4 and PD-L1 (20x). **d, f, h.** Quantitative analysis of images stained by the DAB method.
TILs, tumour-infiltrating lymphocytes.

ROC curve

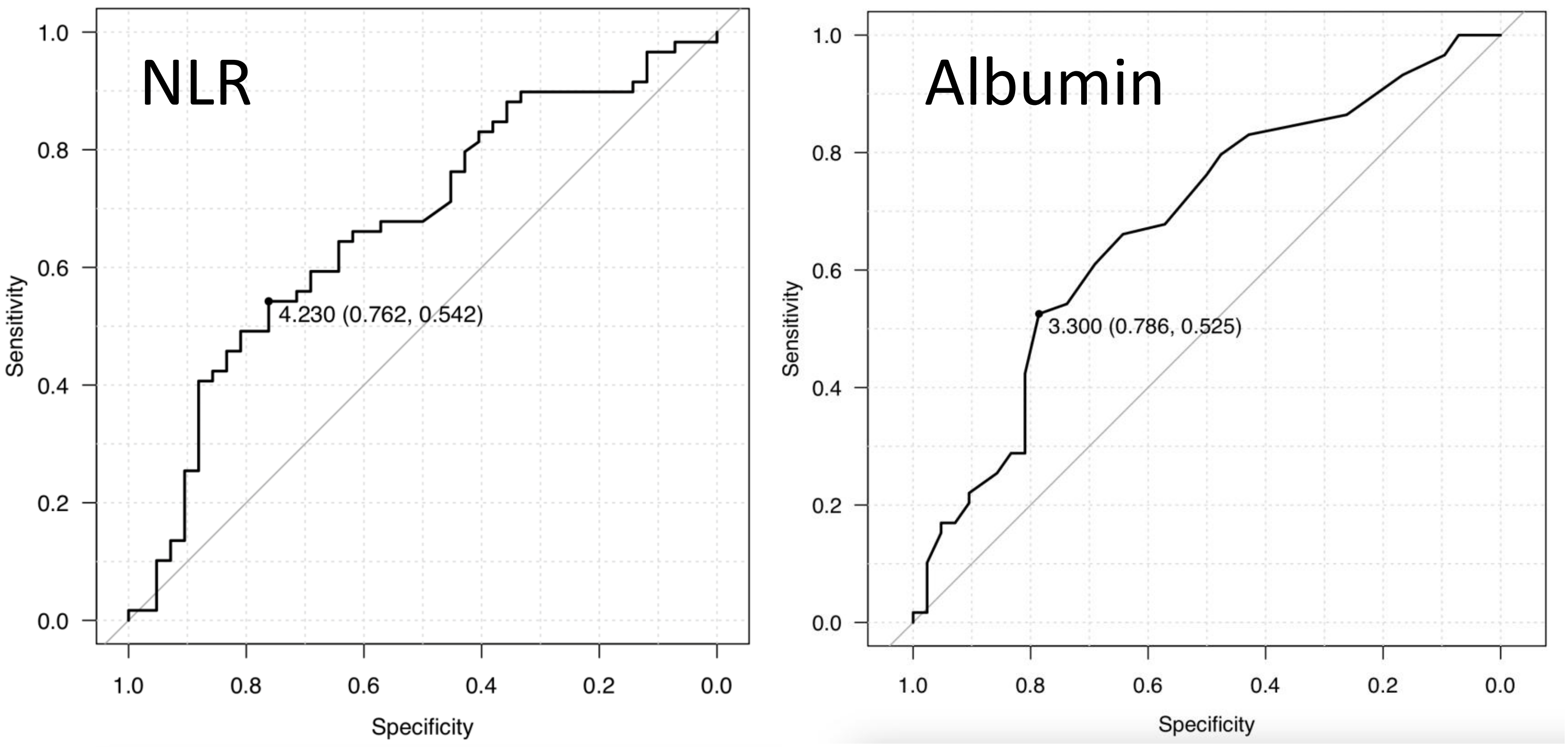

	Range	Median	Cut off	Area Under the ROC Curve(95%CI)
**NLR**	0.93-28.25	3.56	4.23	0.6697(0.5615-0.7779)
**Albumin(g/dL)**	1.8-4.5	3.6	3.30	0.677 (0.5695-0.7844)

Figure S2:
ROC curves for NLR and albumin.
TILs, tumour-infiltrating lymphocytes; c, combined (intraepithelial + stromal); i, intraepithelial; s, stromal.

ROC curve

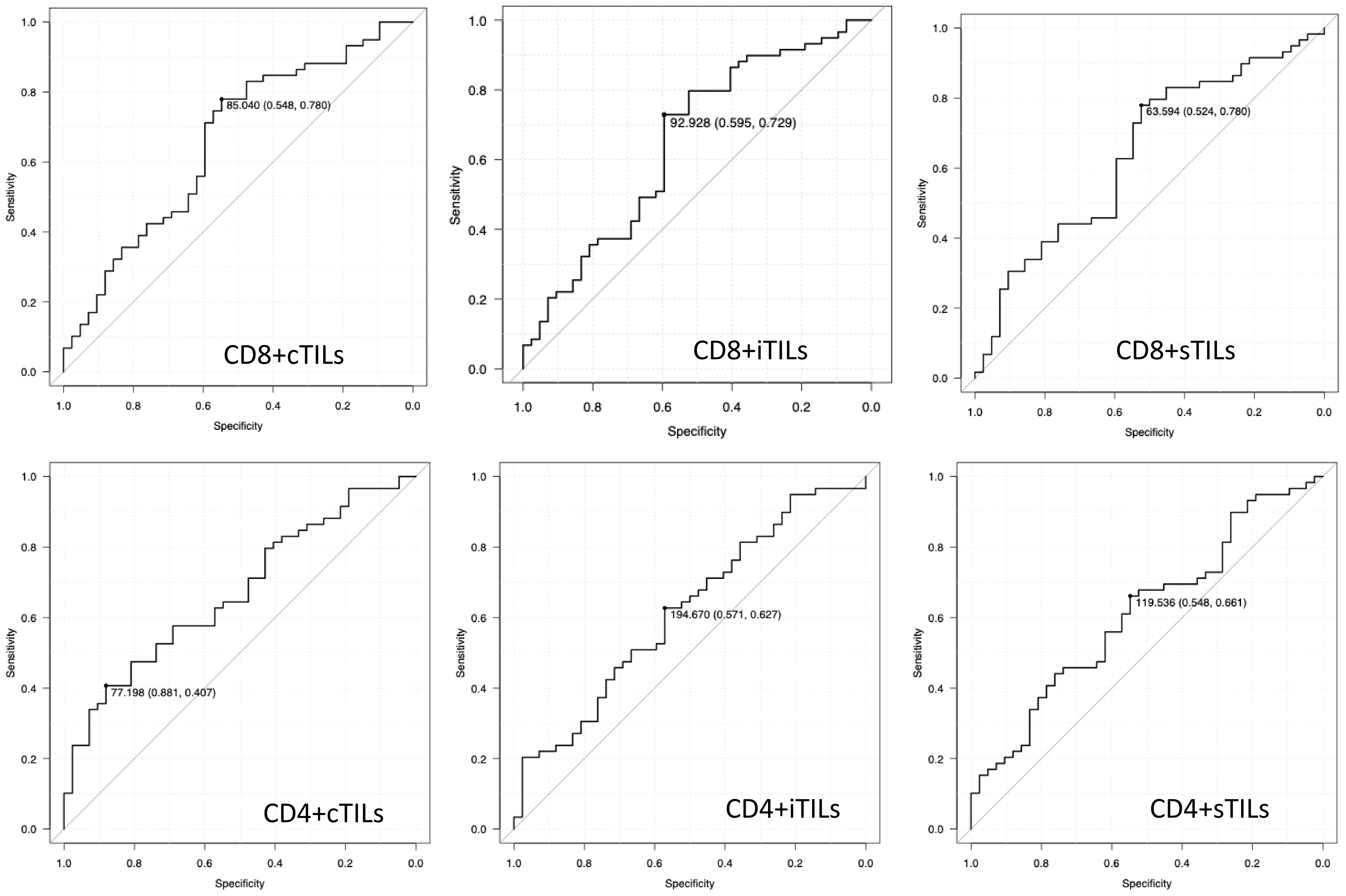

	Range	Median	Cut off	Area Under the ROC Curve(95%CI)
**CD8+cTILs(/mm2)**	3.05-751.8	66.5	85.0	0.6562(0.5461-0.7663)
**CD8+iTILs(/mm2)**	6.22-383.7	67.8	92.9	0.6489(0.5372-0.7606)
**CD8+sTILs(/mm2)**	1.67-726.2	43.9	63.4	0.6421(0.5313-0.7528)
**CD4+cTILs(/mm2)**	0.88-4931.3	151.7	77.2	0.6703(0.5654-0.7752)
**CD4+iTILs(/mm2)**	0.08-2042.5	167.2	194.7	0.6158(0.5045-0.7271)
**CD4+sTILs(/mm2)**	0.10-5536.2	105.8	119.5	0.6134(0.5024-0.7244)

Figure S3:
ROC curves of CD8+/CD4+ cTIL, iTILs and sTILs.
TILs, tumour-infiltrating lymphocytes; c, combined (intraepithelial + stromal); i, intraepithelial; s, stromal.

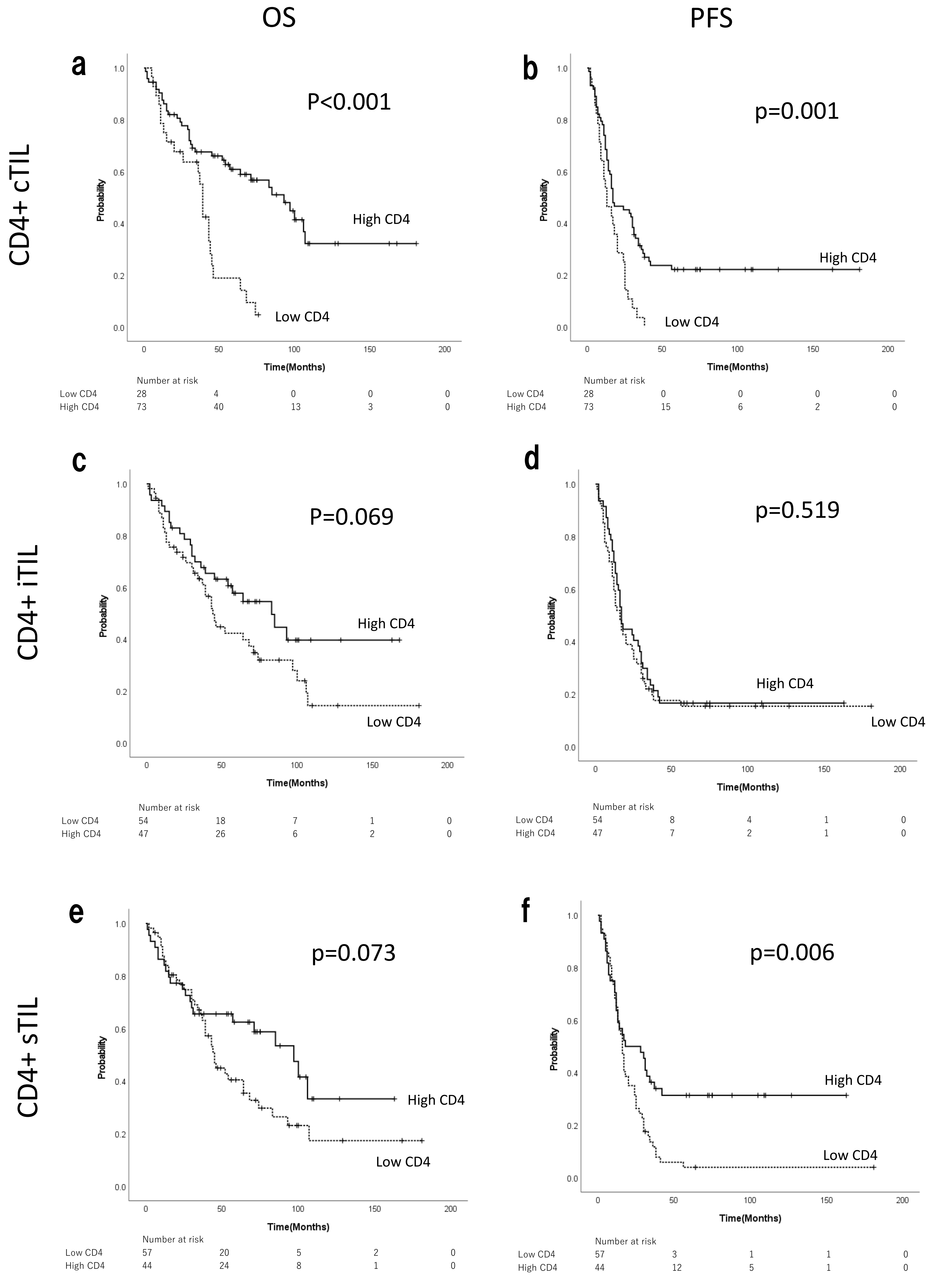

FigureS4:
Kaplan–Meier curves for OS and PFS with EOC stratified by CD4+ cTILs (a and b), iTILs(c and d) and sTILs (e and f). OS, overall survival; PFS, progression-free survival; TILs, tumour-infiltrating lymphocytes; c, combined (intraepithelial + stromal); i, intraepithelial; s, stromal.

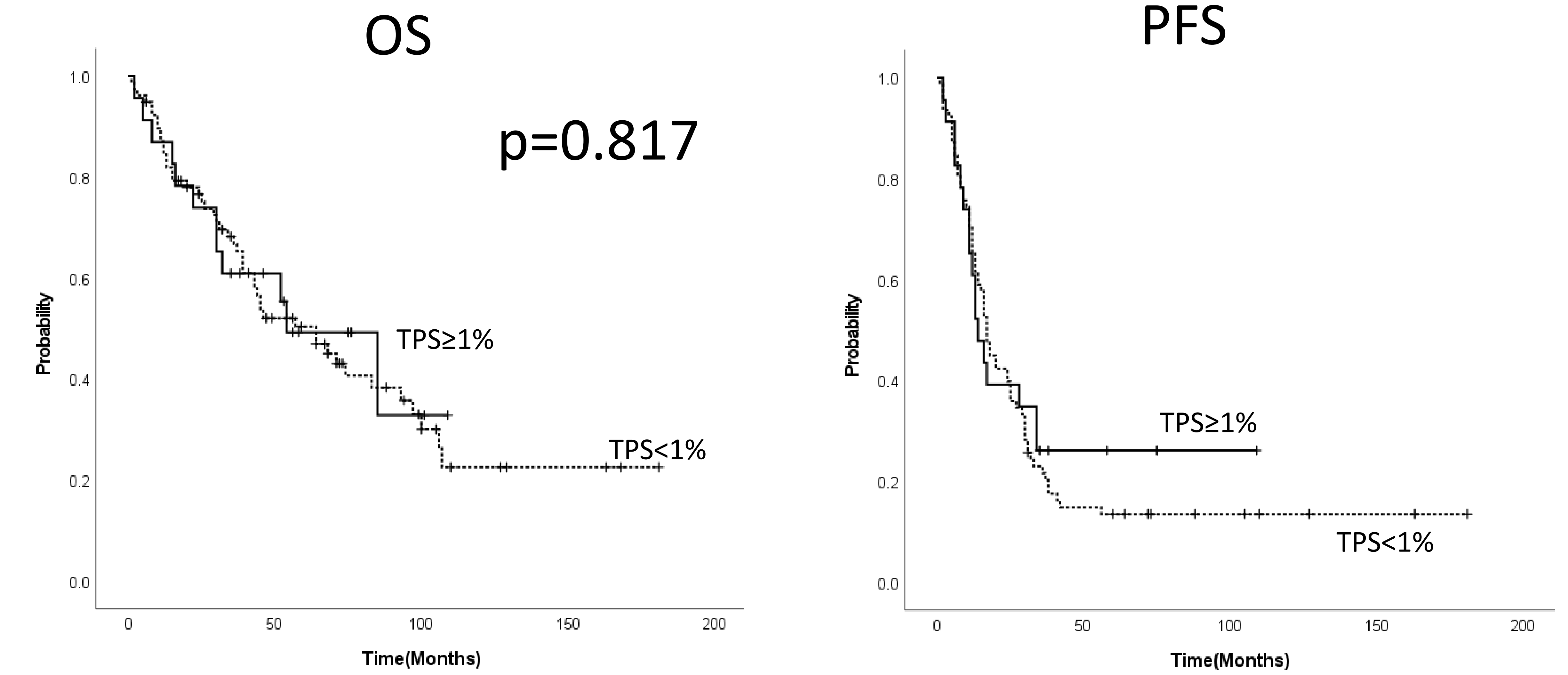

	Number at risk				
TPS<1%	78	33	11	3	0
TPS≥1%	23	11	2	0	0

	Number at risk				
TPS<1%	78	11	5	2	0
TPS≥1%	23	4	1	0	0

Figure S5:
Kaplan–Meier curves for OS and PFS with epithelial ovarian cancer stratified by TPS.
OS, overall survival; PFS, progression-free survival; TPS, tumour proportion score.

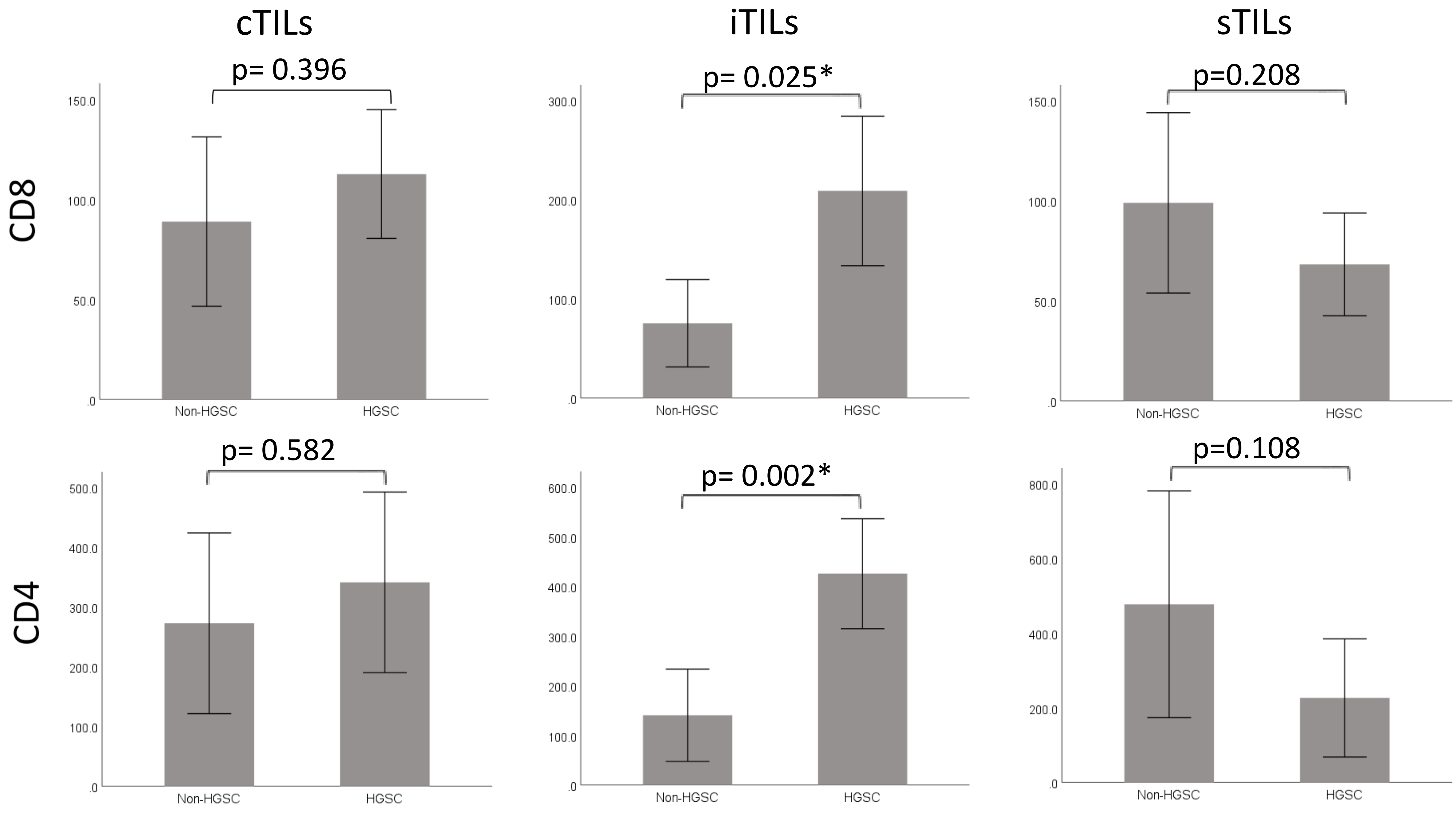

FigureS6:
Density of CD8+ and CD4+ TILs in HGSC and in non HGSC; the other histological subtypes (clear cell, endometrioid, and mucinous carcinoma).
HGSC, high-grade serous carcinoma; TILs, tumour-infiltrating lymphocytes; c, combined (intraepithelial + stromal); i, intraepithelial; s, stromal.

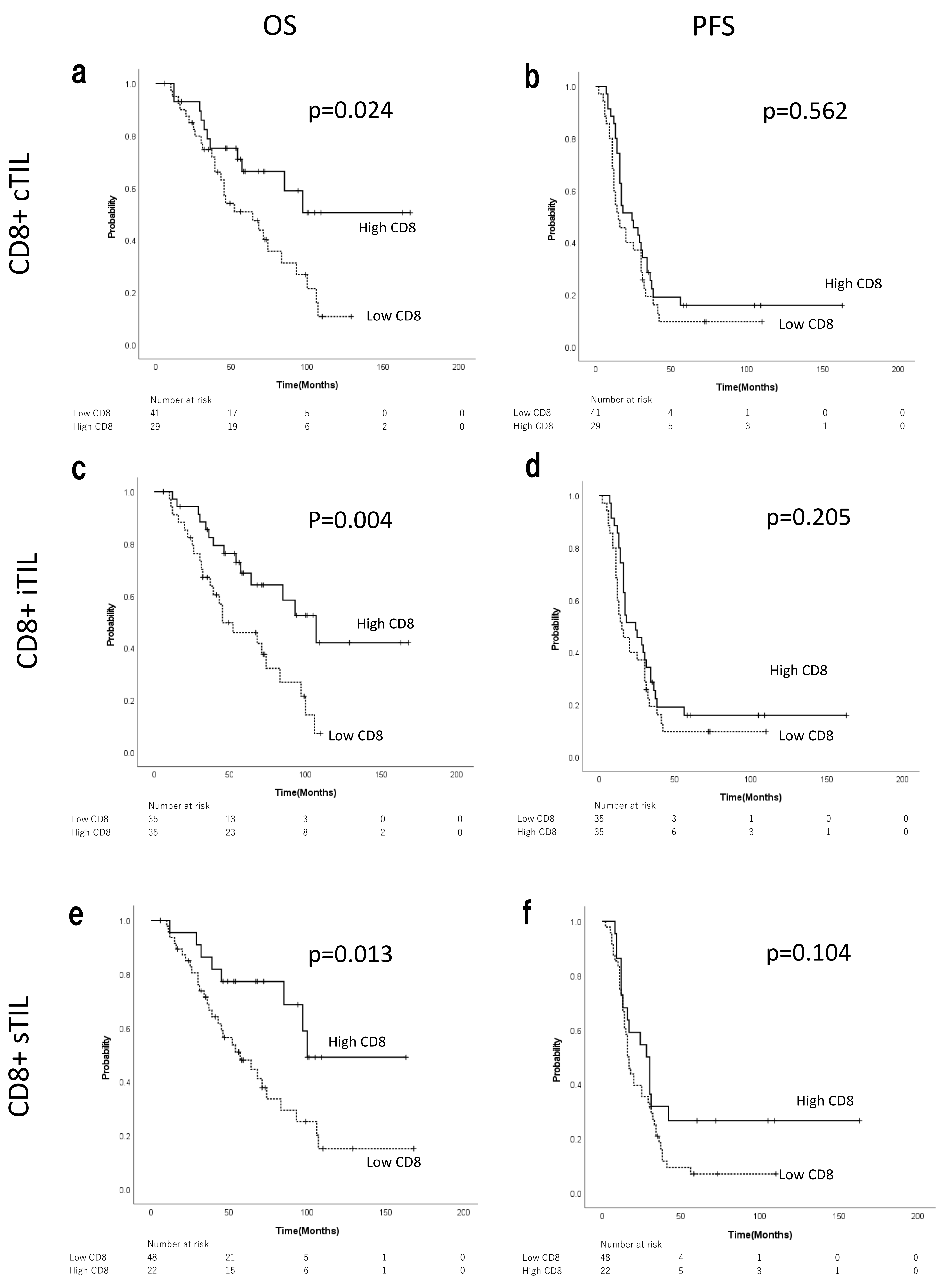

FigureS7:

Kaplan–Meier curves for OS and PFS with HGSC stratified by CD8+ cTILs (a and b), iTILs(c and d) and sTILs (e and f). OS, overall survival; PFS, progression-free survival; HGSC, high-grade serous carcinoma; TILs, tumour-infiltrating lymphocytes; c, combined (intraepithelial + stromal); i, intraepithelial; s, stromal.
